# The SFT2D2 gene is associated with the autoimmune pathology of schizophrenia in a Chinese population

**DOI:** 10.3389/fneur.2022.1037777

**Published:** 2022-12-21

**Authors:** Duilin Liu, Lin Wu, Hui Wei, Caiyun Zhu, Runhui Tian, Wanwan Zhu, Qi Xu

**Affiliations:** ^1^State Key Laboratory of Medical Molecular Biology, School of Basic Medicine Peking Union Medical College, Institute of Basic Medical Sciences Chinese Academy of Medical Sciences, Beijing, China; ^2^Laboratory of Molecular Diagnostics, Beijing Boren Hospital, Beijing, China; ^3^Neuroscience Center, Chinese Academy of Medical Sciences, Beijing, China; ^4^Mental Health Center, The First Bethune Hospital of Jilin University, Changchun, China

**Keywords:** schizophrenia, GWAS, re-genotying, SFT2D2, autoantibody

## Abstract

**Background:**

The relative risk of GWAS-confirmed loci strongly associated with schizophrenia may be underestimated due to the decay of linkage disequilibrium between index SNPs and causal variants. This study is aimed to investigate schizophrenia-associated signals detected in the 1q24-25 region in order to identify a causal variant in LD with GWAS index SNPs, and the potential biological functions of the risk gene.

**Methods:**

Re-genotyping analysis was performed in the 1q24-25 region that harbors three GWAS index SNPs associated with schizophrenia (rs10489202, rs11586522, and rs6670165) in total of 9801 case-control subjects of Chinese Han origin. Circulating autoantibody levels were assessed using an in-house ELISA against a protein derived fragment encoded by SFT2D2 in total of 682 plasma samples.

**Results:**

A rare variant (rs532193193) in the SFT2D2 locus was identified to be strongly associated with schizophrenia. Compared with control subjects, patients with schizophrenia showed increased anti-SFT2D2 IgG levels. Receiver operating characteristic (ROC) analysis revealed an area under the ROC curve (AUC) of 0.803 with sensitivity of 28.57% against specificity of 95% for the anti-SFT2D2 IgG assay.

**Discussion:**

Our findings indicate that SFT2D2 is a novel gene for risk of schizophrenia, while endogenous anti-SFT2D2 IgG may underlie the pathophysiology of the immunological aspects of schizophrenia.

## Introduction

Schizophrenia (SCZ) is a severely debilitating disorder, affecting 0.7–1.1% of the population worldwide (1). The etiology of SCZ is complicated, wherein genetic factors contribute to susceptibility with an estimated heritability of up to 80% (2, 3).

The genome-wide association studies (GWASs) over the past decade draw a blueprint for the heritability of SCZ, with hundreds of loci strongly associated with SCZ (*P* < 5 × 10^−8^) (4– 6). Yet, GWAS on SCZ gave us some important tips: (1) most loci of small effect (odds ratio, OR < 1.3 generally) contribute to the etiology of SCZ; (2) associations were concentrated in genes of the central nervous system and implicated fundamental processes related to neuronal function (6); and (3) variations enriched at HLA and B-cell activation pathways support for an association between immune system abnormalities in the pathology of some patients with SCZ (7). It is indisputable that some potential genes and pathways involved in schizophrenia are shared across populations (8). Sampling different ancestries can provide insights into specific patterns of linkage disequilibrium (LD) and key single-nucleotide polymorphisms (SNPs) in this region (8–10). However, others may be population specific, and the heterogeneity related to ancestry will produce different causal variants and LD patterns. For example, one of the most consistent findings in schizophrenia GWAS studies in European populations is the association with the major histocompatibility complex (MHC) region, but GWAS of Asian populations usually fail to find evidence (8, 11). The most recent GWAS on SCZ conducted by PGC reported common variant associations at 287 distinct genomic loci in over 320,000 individuals, but most of the participants were of European descent, with less than 18% East Asia individuals (6). The divergent genetic architecture of schizophrenia across all traits is very likely to have been subject to different evolutionary pressures, and there should be population-specific risk factors.

We reviewed all available GWAS summary data derived from Chinese Han origin SCZ individuals and found that association signals in chromosome 1q24.2 were well repeated across different studies. In this region, rs6670165 was associated with SCZ in both Chinese and European populations (*p* < 5 × 10^−8^) (12), and two nearby loci rs11586522 and rs10489202 were associated with Chinese SCZ (13), although their *p*-values did not meet genome-wide significance. Thus, we hypothesize that rs6670165 may be a leading index signal, and its association signal may come from a causal variant with low frequency in the 1q24-25 region. The Chinese SCZ loci rs11586522 and rs10489202 are useful for the construction of a haplotype to stratify study samples.

First, the present study was undertaken to replicate the allelic association of rs10489202 and rs11586522 with schizophrenia in an independent Chinese sample and to examine whether the effect size of a genetic variant identified by GWAS was underestimated. If this was the case, a causal variant should be enriched in patients who carried a schizophrenia-associated rs10489202–rs11586522 haplotype. Second, a four-step analysis strategy was performed in this study (Supplementary Figure S1): step one was to identify a schizophrenia-associated rs10489202–rs11586522 haplotype, step two was to identify disease-related variants by deep sequencing of a 2-Mb region surrounding rs10489202 and rs11586522 in homozygotic case–control samples that carried two copies of the schizophrenia-associated rs10489202–rs11586522 haplotype, step three was to verify a possible causal variant in a large mixed case–control sample, and step four was to verify the causal variant in an independent sample. Finally, for the identified risk genes, their possible roles in the pathogenesis of schizophrenia will be clarified through bioinformatics prediction and biological function detection.

## Materials and methods

### Participants

Three sets of independent case–control samples of Chinese Han origin were collected between 2008 and 2018 in this study. The sample size and demographic features of each set are listed in Supplementary Table S1. The set 1 and set 2 DNA samples were derived from the study by Shi Y et al. as reported previously (13); they were used for genetic analysis only. All patients in set 3 were drug-free, and first-episode schizophrenia was recruited in the First Bethune Hospital of Jilin University between 2016 and 2018, while healthy controls were randomly selected from volunteers who were requested to reply to a written invitation to evaluate their medical history. All the patients were diagnosed according to the criteria of the Diagnostic and Statistical Manual of mental disorders, fourth edition (DSM-IV) for schizophrenia. The participants met all the inclusion criteria and exclusion criteria (Supplementary Table S2). All participants provided written informed consent to attend this study as approved by the Ethics Committee of the Chinese Academy of Medical Science and Peking Union Medical College and conformed to the requirements of the Declaration of Helsinki.

### Sample processing

Plasma was separated by 2,000 rpm centrifugation for 15 min, aliquoted to 200 μl/tube, and stored at −80°C until assay. Genomic DNA was extracted from the whole blood sample using the QIAamp DNA Mini Kit (Qiagen, Shanghai, China) according to the manufacturer's instructions. DNA purity was assessed by measuring the A260/A280 ratio (1.8–2.0) using NanoDrop2000c (Thermo Scientific, Shanghai, China) and DNA quality was checked by 0.8% agarose gel electrophoresis for a strong band at a high molecular weight (>10 kb).

### Genotyping

#### Target regional sequencing and MALDI-TOF-MS genotyping

Forty-eight samples (24 cases and 24 controls) that carried two copies of the rs10489202–rs11586522 haplotype underwent deep sequencing in a 2-Mb region surrounding rs10489202 and rs11586522 (chr1:166997055–168997055, hg19) using the HaloPlex Target Enrichment system (Agilent, Santa Clara, USA). In brief, genomic DNA samples were digested by a cocktail of restriction enzymes and hybridized to specific probes incorporating Illumina-specific sequence motifs. Hybridized molecules were captured with magnetic beads, subsequently amplified using indexed primers, and finally sequenced with the HiSeq2000 system (Novogene Bioinformatics Technology Co Ltd, Beijing, China). The variants that showed disease association were validated with Sanger sequencing to exclude false positive signals. Positive loci were then genotyped with matrix-assisted laser desorption ionization time-of-flight mass spectrometry (MALDI-TOF-MS) using the MassARRAY iPLEX system (Sequenom, Inc, San Diego, California, USA) in all individuals who carried at least one copy of the rs10489202–rs11586522 haplotype of interest, namely the validation sample. The primers used for PCR reaction and template-directed single-base extension were designed using the MassARRAY Assay Design 3.1 software based on the instructions of the manufacturer (Supplementary Table S3). Genotyping 5% positive samples maintained quality control, which was performed blindly by the technical personnel, and all duplicates were congruent.

#### TaqMan genotyping assay

A TaqMan SNP genotyping assay was performed using FastStart Essential DNA Probes Master (Roche, Mannheim, Germany) on the LightCycler^®^ 96 system (Roche) for all SNPs except rs6670165, according to the manufacturer's instructions. The information for primers and probes is listed in Supplementary Table S4.

#### PCR-based restriction fragment length polymorphism analysis

There is a MluCI restriction site on the DNA sequence of rs6670165, which may affect TaqMan genotyping. We, thus, design a PCR-based restriction fragment length polymorphism assay for rs6670165 genotyping (Supplementary Table S4). The PCR products were 133 base pairs (bp) in length and contained two MluCI sites, of which one was constant and produced a 6-bp fragment in both alleles of rs6670165, so that the complete digestion of the rs6670165T allele by MluCI restriction endonuclease produced two fragments of 29 and 98bp, respectively. The genotype call was performed manually on capillary gel electrophoreses.

### Detection of plasma anti-SFT2D2 IgG levels

The computational prediction of the HLA-II restricted epitopes was performed using the Immune Epitope Database (http://www.iedb.org/) and an N-terminal linear peptide of SFT2D2 (KLKKVLSGQDTEDRSGLSEVVEAS) displayed excellent predicted scores above the threshold (Supplementary Figure S2). A previously reported in-house enzyme-linked immunosorbent assay (ELISA) was optimized for plasma anti-BICD2 IgG detection (14). The linear peptide antigen was synthesized by solid-phase chemical method with a purity of > 95% and dissolved in 67% acetic acid to obtain a 5 mg/ml stock solution (stored at −20°C). The working solution was diluted just before use with coating buffer (0.1 M phosphate buffer containing 0.15 M NaCl and 1 mM EDTA) to 10 μg/ml. Maleimide-activated 96-well microtiter plates (CLS2510-50EA/CLS2509-50EA, Sigma-Aldrich, Shanghai, Beijing) were coated in 100 μl/well of the antigen and incubated for 90 min at 37°C. Plates were washed three times using 200 μl of wash buffer A (0.1 M phosphate buffer containing 0.15 M NaCl) and blocked using 200 μl (10 μg/ml) of cysteine/well in coating buffer for 1 h at room temperature. Plates were washed two times with 200 μl of wash buffer A and dried at 40°C for 3 h. Dried plates were sealed with sealing film and stored at 4°C until use. The plates were washed two times with 200 μl of wash buffer B (phosphate-buffered saline [PBS] containing 0.1% Tween-20) in each well to rehydrate. The plasma sample (including a positive control, PC) was diluted 1:100 in assay buffer (PBS containing 0.5% bovine serum albumin) and 50 μl of the sample was loaded into each sample well; 50 μl of assay buffer was added to each negative control (NC) well. Following incubation at 37 °C for 120 min, the plate was washed three times with 200 μl of wash buffer B and 50 μl of peroxidase-conjugated goat anti-human IgG Fc (ab98624, Abcam, Cambridge, UK) diluted 1:40000 in assay buffer was then added and incubated for 60 min at room temperature. After incubation, the plate was washed three times with 200 μl of wash buffer B, then 50 μl of 3,3′,5,5′-tetramethylbenzidine (PR1200, Solarbio, Beijing, China) was added, and the plate was incubated in the dark for 20 min before 50 μl of the stop solution was added (C1058, Solarbio, Beijing, China).

The optical density (OD) of each well was measured within 10 min with a plate reader at 450 nm with a reference wavelength of 620 nm. All samples were tested in duplicate and the specific binding ratio (SBR) was calculated for each sample using the following formula:


$$\begin{array}{l} SBR\;=\;\left(OD_{sample}\;-\;OD_{NC}\right)/\left(OD_{PC}\;-\;OD_{NC}\right) \end{array}$$

### Statistical analysis

The genotypic distributions of all SNPs studied were examined by the chi-square (χ2) goodness-of-fit test for Hardy–Weinberg equilibrium (HWE). UNPHASED (Version 3.1.7, Cambridge, UK) was applied to analyze the genotyping data for allelic and haplotypic associations, with the calculation of OR values and 95% confidence interval (CI). For the sequence capture data, UnifiedGenotyper of GATK (https://software.broadinstitute.org/gatk/) to call SNP/INDEL was applied to identify the difference in DNA sequence between case and control samples, and the Plink (http://pngu.mgh.harvard.edu/~purcell/plink/) was then applied to analyze the disease association signal at each variant of interest in 48 homozygotic case–control samples as mentioned above. All target DNA sequences used in this study were retrieved from the hg19 database.

Statistical analysis was performed using GraphPad Prism (Version 9.0.0, GraphPad Software, CA, USA). Mann–Whitney *U*-test was used to examine the differences in plasma anti-SFT2D2 IgG levels. Receiver operating characteristic (ROC) analysis was applied to work out the area under the ROC curve (AUC) with a 95% confidence interval (CI) and the sensitivity of the ELISA antibody test against a specificity of ≥95%.

## Results

### Reanalysis of GWAS-confirmed loci on chr1q24-25

We first searched GWAS-confirmed schizophrenia-associated loci in the Chinese Han population, which are neighboring enough and are useful for the construction of a haplotype to stratify study samples. There were three index SNPs distributed in an area of ~9 Mb located on chr1q24-25: rs10489202 (13) (NC_000001.10:g.167903079G > T), rs11586522 (13) NC_000001.10:g.168091031C > A), and rs6670165 (12) (NC_000001.10:g.177280121C > T), and all were in HWE among the study population (Table 1). Genotyping analysis was conducted in the set-one samples (4125 SCZ vs. 3608 CTL); the disease association signal was detected at rs10489202 (χ2 = 6.27, *P* = 0.012, OR = 1.11, 95% CI 1.02–1.22) and rs6670165 (χ2 = 5.99, *P* = 0.014, OR = 1.13, 95% CI 1.03–1.25) but not at rs11586522 (χ2 = 1.63, *P* = 0.202, OR = 1.05, 95% CI 0.97–1.14) (Table 2). The rs10489202T–rs11586522A haplotype displayed the most significant association (χ^2^ = 7.31, *P* = 6.84 × 10^−3^, OR = 1.14, 95% CI 1.04–1.24), suggesting a potential causal variant for schizophrenia within this region (Table 3).

**Table 1:** Heterozygosity and the Hardy–Weinberg equilibrium.

**SNP**	**Het (Obs)**	**Het (Exp)**	**HWE (*P*)**
rs10489202	0.254	0.254	0.948
rs11586522	0.292	0.301	0.079
rs863454	0.106	0.102	0.085
rs532193193	0.017	0.017	0.627
rs6670165	0.250	0.251	0.940

SNP, single-nucleotide polymorphism; Het, heterozygosity; Obs, observed; Exp, expected; HWE, Hardy-Weinberg equilibrium.

**Table 2:** Association of rs10489202, rs11586522, and rs6670165 with schizophrenia.

**All samples** **(*****n*** = **7700)**		**rs10489202**					**rs11586522**					**rs6670165**				
		**Allele**		**Genotype**			**Allele**		**Genotype**			**Allele**		**Genotype**		
	**G**	**T**	**GG**	**GT**	**TT**	**C**	**A**	**CC**	**CA**	**AA**	**C**	**T**	**CC**	**CT**	**TT**
SCZ	*N* (%)	6913(83.8)	1337(16.2)	2925(70.9)	1063(25.8)	137(3.3)	6629(80.4)	1621(19.6)	2668(64.8)	1293(31.4)	154(3.7)	5755(83.7)	1123(16.3)	2420(70.4)	915(26.6)	104(3.0)
CTR	*N* (%)	6096(85.3)	1054(14.7)	2622(73.3)	852(23.8)	101(2.8)	5847(81.2)	1357(18.8)	2383(66.2)	1081(30.0)	138(3.8)	4463(85.3)	769(14.7)	1937(74.0)	589(22.5)	90(3.4)
	OR		1.12		1.12	1.22		1.05		1.07	1		1.13		1.24	0.92
	95%CI		1.03–1.22		0.50–1.24	0.94–2.37		0.97–1.14		0.48–1.18	0.79–1.26		1.03–1.25		0.55–1.40	0.35–1.23
	χ^2^		6.27		4.42	2.14		1.63		1.75	0		5.99		12.78	0.28
	*p*		0.012		0.036	0.144		0.202		0.186	0.978		0.014		3 × 10^−4^	0.6

SNP, single-nucleotide polymorphism; SCZ, schizophrenia; CTR, control; OR, odds ratio.

**Table 3:** Association of rs10489202–rs11586522 haplotypes with schizophrenia.

	**SCZ**	**CTR**				
**Haplotype**	***N* (%)**	***N* (%)**	**OR**	**95%CI**	**χ^2^**	***P* ^a^**
G-C	6489(78.7)	5682(79.6)	0.94	0.87–1.02	2.08	0.149
G-A	424(5.1)	402(5.6)	0.91	0.79–1.04	1.83	0.176
T-C	140(1.7)	126(1.8)	0.96	0.75–1.22	0.10	0.746
**T-A**	**1197(14.5)**	**928(13.0)**	**1.14**	**1.04–1.24**	**7.31**	**6.84 × 10** ^**−3**^ ^b^

a Global χ^2^ = 8.41, df = 3, *P* = 0.038. ^b^The significance survived Bonferroni correction (*P* < 0.05). SNP, single–nucleotide polymorphism; SCZ, schizophrenia; CTR, control; OR, odds ratio. The rs10489202T-rs11586522A haplotype displayed the most significant association with schizophrenia.

### Target regional sequencing surrounding the rs10489202T–rs11586522A haplotype

Target regional sequencing was conducted in 48 case–control samples that carried two copies of the rs10489202T–rs11586522A haplotype. A total of 48,968 amplicons were analyzed in a 1.91-Mb sequenceable region, with a target coverage of 94.9%. In total, 28 variants with a frequency of >4% in 24 case samples and 0% in 24 control samples were defined as a variant of interest (Table 4) and then validated using the MALDI-TOF-MS genotyping system in 286 case–control samples that carried at least one copy of the rs10489202T–rs11586522A haplotype. The genotype call rate for these variants was 94% on average, and two out of 28 eligible variants displayed disease associations (*P* < 0.05, OR > 2) in the validation sample (Table 4), including rs863454 and rs532193193.

**Table 4:** Twenty–eight variations discovered by deep sequencing.

				**capturing results**			**Sequenom validation**			
**chr**	**position**	**variation**	**minor allele/ major allele**	**case/control**	**OR**		**case/control**	**OR**	**95% CI**	***P* ^a^**
1	167242634	rs374525798	T/C	2/0	–		2/1	1.47	0.13–16.36	0.789
1	167343488	rs532966862	A/G	2/0	–		5/1	3.80	0.44–32.77	0.373
1	167458047		C/T	2/0	–		6/2	2.09	0.42–10.47	0.578
1	167475871	rs1799704	C/A	5/0	–		27/18	1.22	0.66–2.28	0.525
1	167475885	rs7534569	A/C	5/0	–		19/11	1.49	0.69–3.20	0.303
1	167482045	rs858551	G/C	5/0	–		25/13	1.43	0.71–2.86	0.312
1	167488799	rs863454	A/C	5/0	–		26/8	2.75	1.22–6.19	0.011
1	167562802	rs548135867	C/T	2/0	–		3/2	1.08	0.18–6.52	0.711
1	167571678	rs539465026	G/A	2/0	–		10/4	1.49	0.46–4.81	0.506
1	167743526	rs568146973	C/T	2/0	–		8/1	6.77	0.84–54.48	0.082
1	167825485	rs2071921	T/C	6/0	–		46/22	1.64	0.96–2.81	0.071
1	167863359	rs532020960	G/T	2/0	–		4/3	0.98	0.22–4.44	0.715
1	167923123		T/C	2/0	–		4/0	–	–	0.195
1	167935501		T/A	2/0	–		4/6	0.51	0.14–1.83	0.467
1	167993356		G/A	3/0	–		44/74	0.34	0.23–0.53	4.22 × 10^−7^
1	168095936	rs564160350	T/C	2/0	–		10/12	0.59	0.25–1.38	0.216
1	168117597	rs533350603	A/G	2/0	–		2/2	0.77	0.11–5.51	0.806
1	168122619	rs543569784	T/C	7/0	–		22/9	2.00	0.90–4.42	0.082
1	168206831	rs532193193	A/T	2/0	–		7/0	–	–	0.045
1	168207770		G/T	2/0	–		4/0	–	–	0.191
1	168213437		G/A	2/0	–		5/2	1.85	0.36–9.63	0.720
1	168251318	rs566388016	T/C	2/0	–		2/2	0.72	0.10–5.18	0.853
1	168259441	rs529952842	T/C	2/0	–		11/3	2.79	0.77–10.12	0.104
1	168262071	rs377343745	T/C	4/0	–		8/2	3.30	0.69–15.69	0.205
1	168302624	rs571065842	T/G	2/0	–		2/3	0.45	0.07–2.71	0.662
1	168402778		C/T	2/0	–		2/0	–	–	0.642
1	168603791	rs528912102	A/G	2/0	–		4/4	0.74	0.18–2.97	0.942
1	168722434		G/A	2/0	–		2/1	1.47	0.13–16.36	0.789

chr, chromosome; OR, odds ratio referring to minor allele. ^a^If the expected allele number was between 1 and 5, a continuity correction was performed to recalculate *p*-value. All coordinates are given in hg19.

### Genotyping verification of rs863454 and rs532193193

Further genotyping of these two SNPs with the set-one samples revealed that rs532193193 was the only SNP showing a strong association with schizophrenia (χ2 = 8.86, *P* = 2.92 × 10^−3^, OR = 1.65, 95% CI = 1.18–2.30) (Table 5). The results were repeated in the set-two samples (χ^2^ = 3.60, *P* = 0.058, OR = 1.75, 95% CI = 0.98–3.13); combined results of two sample sets displayed a stronger association for rs532193193 (χ^2^ = 12.33, *P* = 4.47 × 10^−4^, OR = 1.67, 95% CI 1.25–2.22, Table 6).

**Table 5:** Association of two SNPs of interest with schizophrenia.

**All samples (*****n*** = **7200)**		**rs863454**					**rs532193193**				
		**Nearest gene(s):** ***AKR1D1P1***					**Nearest gene(s):** ***SFT2D2***				
		**Allele**		**Genotype**			**Allele**		**Genotype**		
		**C**	**A**	**CC**	**CA**	**AA**	**T**	**A**	**TT**	**TA**	**AA**
SCZ	*N* (%)	6663(94.2)	411(5.8)	3167(89.5)	329(9.3)	41(1.2)	7049(98.6)	101(1.4)	3476(97.2)	97(2.7)	2(0.1)
CTR	*N* (%)	5719(94.6)	327(5.4)	2720(90.0)	279(9.2)	24(0.8)	6210(99.1)	54(0.9)	3078(98.3)	54(1.7)	0(0.0)
	OR		1.08		1.01	1.47		1.65		1.59	–
	95% CI		0.93–1.25		0.86–1.2	0.89–3.65		1.18–2.30		1.14–3.34	0.72–
	χ^2^		0.99		0.02	2.23		8.86		7.44	1.77
	*p*		0.32		0.88	0.14		2.92 × 10^−3^		6.4 × 10^−3^	0.18

SNP, single-nucleotide polymorphism; SCZ, schizophrenia; CTR, control; OR, odds ratio.

**Table 6:** Allelic association of rs532193193 with schizophrenia in two sets of samples.

**All samples (*****n*** = **9711)**		**Set-one**					**Set-two**					**Combined**				
		**Allele**		**Genotype**			**Allele**		**Genotype**					**Genotype**	
		**T**	**A**	**TT**	**TA**	**AA**	**T**	**A**	**TT**	**TA**	**AA**	**T**	**A**	**TT**	**TA**	**AA**
SCZ	*N* (%)	7049(98.6)	101(1.4)	3476(97.2)	97(2.7)	2(0.1)	1904(98.4)	30(1.6)	938(97.0)	28(2.9)	1(0.1)	8953(98.6)	131(1.4)	4414(97.2)	125(2.7)	3(0.1)
CTR	*N* (%)	6210(99.1)	54(0.9)	3078(98.3)	54(1.7)	0(0.0)	2004(99.1)	18(0.9)	993(98.2)	18(1.8)	0(0.0)	8214(99.1)	72(0.9)	4071(98.3)	72(1.7)	0(0.0)
	OR		1.65		1.59	–		1.75		1.65	–		1.67		1.6	–
	95% CI		1.18–2.30		1.14–3.34	0.72–		0.98–3.13		0.92–3.07	0.12–		1.25–2.22		1.20–3.22	0.49–
	χ^2^		8.86		7.44	1.77		3.6		2.72	1.06		12.32		10.08	2.77
	*P*		2.92 × 10^−3^		6.4 × 10^−3^	0.18		0.058		0.1	0.3		4.47 × 10^−4^		1.5 × 10^−3^	0.1

SCZ, schizophrenia; CTR, control; OR, odds ratio.

### Analysis of the rs532193193-containing haplotypes

SNP rs532193193 was used to construct two haplotype systems with the rs10489202–rs11586522 haplotypes and rs6670165. Of the six individual rs10489202–rs11586522–rs532193193 haplotypes (Table 7), the T-A-A haplotype was strongly associated with the risk of schizophrenia (χ^2^ = 16.62, *P* = 4.56 × 10^−5^, OR = ∞). Of the four individual rs6670165–rs532193193 haplotypes (Table 8), the T-A haplotype displayed a strong association with the disease (χ^2^ = 8.00, *P* = 4.70 × 10^−3^, OR = 4.14, 95% CI =1.55–11.09). Haplotype analysis demonstrated that rs532193193 appeared to contribute to the association signal detected at rs10489202, rs11586522, and rs6670165.

**Table 7:** Haplotypic association of the rs10489202–rs11586522–rs532193193 haplotypes with schizophrenia.

**Haplotype**	**SCZ *N* (%)**	**CTR *N* (%)**	**OR**	**95%CI**	**χ^2^**	**P^a^**
G-C-T	5533(77.4)	4926(78.9)	0.91	0.84–1.00	4.28	0.039
G-C-A	82(1.1)	54(0.9)	1.33	0.94–1.88	2.60	0.104
G-A-T	352(4.9)	329(5.3)	0.93	0.80–1.09	0.82	0.370
T-C-T	110(1.5)	108(1.7)	0.89	0.68–1.16	0.76	0.384
T-A-T	1054(14.7)	829(13.3)	1.13	1.02–1.25	5.95	0.015
T-A-A	19(0.3)	0(0)	∞	–	16.62	4.56 × 10^−5^ ^b^
Total	7150	6246				

The order of variants forming the haplotype system is as follows: rs10489202–rs11586522–rs532193193. ^a^Global χ^2^ = 18.09, df = 5, *P* = 2.83 × 10^−3^. ^b^The significance survived Bonferroni correction (*P* < 0.05). SCZ, schizophrenia; CTR, control; OR, odds ratio.

**Table 8:** Haplotypic association of the rs6670165–rs532193193 haplotypes with schizophrenia.

**Haplotype**	**SCZ *N* (%)**	**CTR *N* (%)**	**OR**	**95%CI**	**χ^2^**	**p^a^**
C-T	5384(81.8)	4371(84.4)	0.83	0.75–0.92	13.86	1.97 × 10^−4*b*^
C-A	77(1.2)	45(0.9)	1.35	0.93–1.95	2.56	0.110
T-T	1096(16.7)	756(14.6)	1.17	1.06–1.29	9.22	2.39 × 10^−3*b*^
T-A	21(0.3)	4(0.08)	4.14	1.55–11.09	8.00	4.70 × 10^−3*b*^
Total	6578	5176				

a Global χ^2^ = 20.01, df = 3, *p* = 1.69 × 10^−4^. ^b^The significance survived Bonferroni correction (*p* < 0.05). SCZ, schizophrenia; CTR, control; OR, odds ratio.

### Functional prediction of *SFT2D2*, the host gene of rs532193193

**Figure 1:**
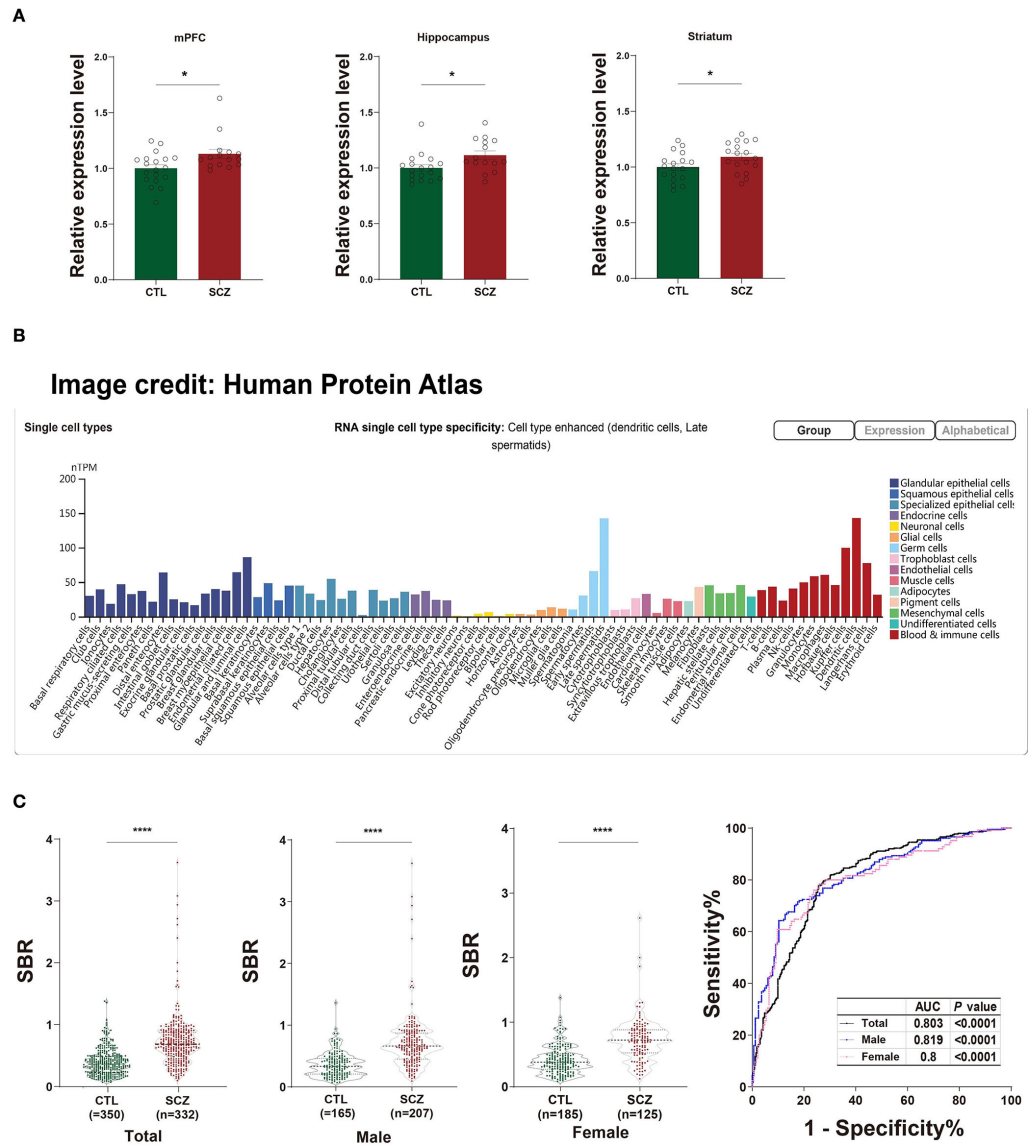
Functional prediction of *SFT2D2*. **(A)** The expression levels of *SFT2D2* mRNA in the postmortem prefrontal cortex, hippocampus, and striatum of the human brain (18 SCZ vs. 15 CTL, from GSE53987 data set) (15). **(B)** *SFT2D2* mRNA levels in single-cell sequencing across 76 cell types from Human Protein Atlas (https://www.proteinatlas.org/ENSG00000213064-SFT2D2/single$+$cell$+$type) (16). *SFT2D2* displayed enhanced distribution in plasmacytoid dendritic cells (pDCs) and late spermatids. **(C)** Anti-SFT2D2 IgG levels in human plasma. Left: SBR values of anti-SFT2D2 IgG levels in combined subjects (350 CTL vs. 332 SCZ), male subjects (165 CTL vs. 207 SCZ), and female subjects (185 CTL vs. 125 SCZ). Right: Receiver operating characteristic curve (ROC) of anti-SFT2D2 IgG assay. Data are expressed as mean ± standard error of the mean (SEM) and individual scatter plots are shown. CTL, control; SCZ, schizophrenia; SBR, specific binding ratio; AUC, area under the ROC curve.

The rs532193193 is located in intron 6 of the *SFT2D2* gene, a vesicle transporter of multipass membranes required for endosome-to-Golgi retrieval. The expression level of *SFT2D2* mRNA increased in postmortem brain samples of patients with schizophrenia, including the pre-frontal cortex, hippocampus, and striatum (18 patients vs. 15 controls, Figure 1A). In single-cell analysis, there were higher *SFT2D2* mRNA levels in peripheral immune cells than neuronal cells (Figure 1B), of which *SFT2D2* was most abundant in pDCs that can rapidly and massively release type I interferon that may trigger autoimmune responses. Taken together, we hypothesized that overactive *SFT2D2* in the brain and pDCs may disrupt self-surveillant homeostasis and trigger autoimmune inflammatory responses in schizophrenia.

### An increase in plasma anti-SFT2D2 IgG levels in schizophrenia

The in-house ELISA developed based on a previous study (17) demonstrated a significant increase in plasma anti-SFT2D2 IgG levels in patients with schizophrenia compared with control subjects (*Z* = −13.686, fold change [FC] = 1.847, *P* < 0.0001) without gender differences (Supplementary Table S5, Figure 1C). ROC analysis revealed that the plasma anti-SFT2D2 IgG assay had an AUC of 0.803 with a sensitivity of 28.57% against a specificity of 95% (Supplementary Table S5, Figure 1C).

## Discussion

Genome-wide association studies have applied a threshold of *P* < 5 × 10^−8^ to claim disease associations that are unlikely to be observed by chance (18). However, this correction may be overly conservative, which is likely to inflate the type-II errors (19). Each index SNP detected in the GWA study has been treated as an independent event, although individual SNPs present in the same chromosomal regions may be in strong LD with one another.

### Are the index SNPs identified by GWA study causal variants?

In the present study, we tested three index SNPs identified by GWA studies (7, 13). While these three SNPs showed weak allelic or haplotypic associations with schizophrenia, their effect size is similar to that reported by the GWA studies, suggesting that our sample size is not large enough to achieve the genome-wise *p*-value that is currently used to claim statistical significance in GWA studies (20). In fact, these three index SNPs themselves are unlikely to play a role in conferring the risk of schizophrenia but suggest that a causal variant may exist around them (21, 22). As a result, the effect size of the causal variant that is rare in the population may be underestimated due to incomplete LD between an index SNP and the causal variant, as well as differences in allele frequency of index SNPs among study populations. Interestingly, the 2-Mb regional sequencing revealed that rs532193193 present in intron 6 of *SFT2D2* (NM_199344.2) was strongly associated with schizophrenia in both the samples that carried the rs10489202T–rs11586522A haplotype and those that did not carry the rs10489202T–rs11586522A haplotype (Tables 4–6). It is possible that rs532193193 is a causal variant in the 1q24-25 region, contributing to the association signal detected at rs10489202, rs11586522, and rs6670165 in GWAS (Tables 7, 8), although an additional causal variant located in the same chromosomal region or nearby cannot be ruled out (23). Generally speaking, heterogeneous samples always affect statistical power for the replication of an association signal that was detected at an index DNA marker in an initial study, especially in large-scale GWAS (24). The causal variant should be enriched in all patients, while different populations may have different index DNA markers in LD with the causal variant. The use of index DNA markers and haplotypes of interest to stratify the population could obtain relatively homogeneous samples and enhance sample power. In our study, the final odd ratio of rs532193193 reached 1.67, which was much stronger than OR values (1.05–1.13) of initial GWAS signals, suggesting a moderate risk with the onset of schizophrenia.

### The possible role of rs532193193

Rare variant rs532193193 is located in intron 6 of *SFT2D2* and its minor allele frequency (MAF) is 0.0006 based on the 1,000 Genomes database (http://browser.1000genomes.org). The ENCODE project shows that rs532193193 is present in the DNase hypersensitive area in some immune cells, such as adult T helper (Th) 0, Th1, and Th2 cells (http://genome.ucsc.edu/), and also in the binding cluster (chr1:168206676–168206911, hg19) of B cell-activating transcription factor (BATF, NM_006399.3) that is very important for the development of B and T lymphocytes (25). The T to A base substitution of rs532193193 can increase free energy and change the secondary structure of rs532193193-containing fragments (Supplementary Figure S3), which may influence the binding of transcription factors.

*SFT2D2* spans 16834bp of DNA and contains eight exons and seven introns; it encodes SFT2 domain-containing protein 2 which has been known to serve as a vesicle transporter of multipass membranes. A recent study has suggested that SFT2D2 requires for endosome-to-Golgi retrieval that may be linked to neurodegenerative diseases (26). SFT2D2 has also been found to be linked to IFN-I signaling that is needed nearly for all innate immune activities in the brain following a non-cytopathic viral infection (27).

An intron in any nucleotide sequence of a gene is removed by RNA splicing during the maturation of the final RNA product. While introns do not encode protein sequences, they can regulate gene expression, alternative splicing (28), and intron-mediated enhancement (29). For example, the genes coding for complements 4A (C4A) and 4B (C4B) are distinguished by the presence or absence of a human endogenous retroviral (HERV) insertion (intron 9) that expands the C4 gene from 14kb to 21kb without changing the C4 protein sequence; the C4-HERV sequence could increase the ratio of C4A to C4B expression, which was found to be strongly associated with schizophrenia (30). Accordingly, it is necessary to test whether rs532193193 present in the intron could influence the expression of *SFT2D2* in a future study. We have observed an increase in *SFT2D2* mRNA expression levels in postmortem brain samples of patients with schizophrenia.

### BATF and immune dysfunction in schizophrenia

A b-cell-activating transcription factor is a nuclear basic leucine zipper protein that belongs to the AP-1/ATF superfamily of transcription factors; it is thought to be a negative regulator of AP-1/ATF transcriptional events. The leucine zipper of this protein mediates dimerization with members of the Jun family of proteins (31). BATF is required for the development of Th9, Th17, T follicular helper, and possibly Th2 cells that are a key point of inflammatory processes (32). BATF is required for the differentiation of Th17 cells that coordinate inflammatory responses in host defense and play a pathogenic role in autoimmune disorders (33). Increasing evidence implicates that the dysfunction of the immune system is involved in the pathogenesis of schizophrenia (34). Many inflammatory markers were shown to be significantly elevated in patients with schizophrenia (35). Enrichment analysis showed that schizophrenia-associated genes were highly expressed in both the brain tissues and immune cells (7).

In fact, the serological tests in our study demonstrated a significant increase in circulating anti-SFT2D2 IgG levels in patients with schizophrenia (FC = 1.847, *P* < 0.0001), with a sensitivity (true positive) of 28.57% against a specificity (true negative) of 95%, suggesting that anti-SFT2D2 IgG may be indicative of a subgroup of schizophrenia. While there was no difference in autoantibody levels between men and women, the discrimination of anti-SFT2D2 IgG was more powerful in male subjects (sensitivity = 39.13%) than in female subjects (sensitivity = 29.60 %). This is in accordance with the clinical observations of schizophrenia. Male patients usually have earlier onset, more severe psychiatric symptoms, and more resistance to anti-psychiatric medications than female patients (36, 37). These differences may arise as a result of the immunological backgrounds associated with sex, which also suggested that sex differences should be taken into consideration for the diagnosis of subgroups and precision treatment of schizophrenia (38, 39). The present study, thus, provides further evidence in support of the speculated link between the immune system and schizophrenia.

### Anti-SFT2D2 IgG may underlie the pathophysiology of the immunological aspects of schizophrenia

The *SFT2D2* gene is widely expressed in human brain regions (Figure 1A). Interestingly, we noticed that the gene is also extensively expressed in immune-related cells, especially dendritic cells (peripheral) and microglia (central) (Figure 1B, Supplementary Figure S4). Furthermore, we performed the spatiotemporal expression pattern analysis using the expression data from the Human Brain Transcriptome (https://hbatlas.org/) showing that the mRNA expression levels of the gene are higher at the early developmental stage (i.e., embryonic and fetal stages) compared with childhood and adulthood stages (Supplementary Figure S5). This suggests that the *SFT2D2* gene identified may have a role in the human brain and also makes it possible to become a candidate pathogenic target. In addition, we examined the expression of the gene in SCZ cases and healthy controls. We found that *SFT2D2* is significantly upregulated in the pre-frontal cortex, hippocampus, and striatum of SCZ cases compared with controls (Figure 1A) in the GSE53987 data set (15). Several studies on people with schizophrenia have found that some patients have increased permeability of the blood–brain barrier (40, 41). This may allow the passage of immune cells and antibodies into the brain, leading to altered neuronal–glial function that in turn may lead to inflammation and schizophrenia psychopathology. Now, the anti-SFT2D2 IgG we found in patients with schizophrenia may have a similar mechanism. On the one hand, circulating anti-SFT2D2 IgG can cross the blood–brain barrier to the brain and lead to targeting of SFT2D2 protein which expresses in the central nervous system, thus contributing to the illness and possibly to schizophrenia pathophysiology. On the other hand, overactive *SFT2D2* in the brain and pDCs may disrupt self-surveillant homeostasis and trigger autoimmune inflammatory responses in schizophrenia. All in all, the increased antibody level reflects a breakdown of immune tolerance to the products of these schizophrenia-associated variants, causing an autoimmune response (42). Alternatively, this could indicate hyperactivity of the immune system as part of the etiology of schizophrenia, which would be concordant with current literature suggesting an increase in pro-inflammatory molecules with increased immune response in those undergoing both acute psychosis and chronic schizophrenia (17).

### Limitations of this study

First, regional capture sequencing cannot achieve 100% genome coverage, implying that there may be more than one causal variant in the 1q24-25 region beyond rs532193193 identified in this study. Second, the frequency of the minor allele of rs532193193 is only 0.3% in East Asian ancestry based on the 1000 Genomes Project phase 3, although it is shown to be 1.17% in the Chinese Han population in this study. The low frequency of rs532193193 leads to a lack of effective eQTL information, and its regulatory effect on *SFT2D2* function needs further study. Third, serological assays against anti-SFT2D2 IgG were only performed in patients with schizophrenia in the acute phase and lacked the trend of changes in antibodies after anti-psychiatric medications. It will be useful to repeat these assays in follow-up cohorts, as well as in other psychiatric diseases, to determine whether abnormalities of SFT2D2-associated humoral immunity are limited to schizophrenia and show a state-dependent feature.

## Conclusion

Disease-associated DNA markers identified by GWA studies may be in strong LD with a causal variant only; genetic heterogeneity is likely to reduce the power for the detection of disease association at each locus of interest. The chromosomal region that contains an index DNA marker needs further analysis with deep sequencing in a genetically homogenous group to shed light on the true causal variant for the disease, which may be the best way to unveil each causal variant predisposing to schizophrenia. Following this theory, our study identified a potential causal gene for the risk of schizophrenia, *SFT2D2*, and its peripheral autoantibody levels were significantly increased in patients with schizophrenia.

## Data availability statement

The raw data supporting the conclusions of this article will be made available by the authors, without undue reservation.

## Ethics statement

The studies involving human participants were reviewed and approved by Ethics Committee of the Chinese Academy of Medical Science and Peking Union Medical College. The patients/participants provided their written informed consent to participate in this study.

## Author contributions

DL, HW, and QX contributed to the experimental design. DL and LW conducted all the genotyping experiments and genomic analysis. RT collected plasma samples. DL and HW conducted all serological tests. DL, CZ, RT, and WZ conducted all statistical analysis. DL, HW, and LW wrote the first draft of the manuscript and contributed to manuscript revision.
