# Supplementary material for: The SFT2D2 gene is associated with the autoimmune pathology of schizophrenia in a Chinese population
Source: Front Neurol. 2022 Dec 21;13:1037777. doi: 10.3389/fneur.2022.1037777 (PMC9810986; doi:10.3389/fneur.2022.1037777)
Supplement: Supplementary file 1 [file Data_Sheet_1.docx]

Supplementary Material

# Supplementary Figures and Tables

## Supplementary Figures


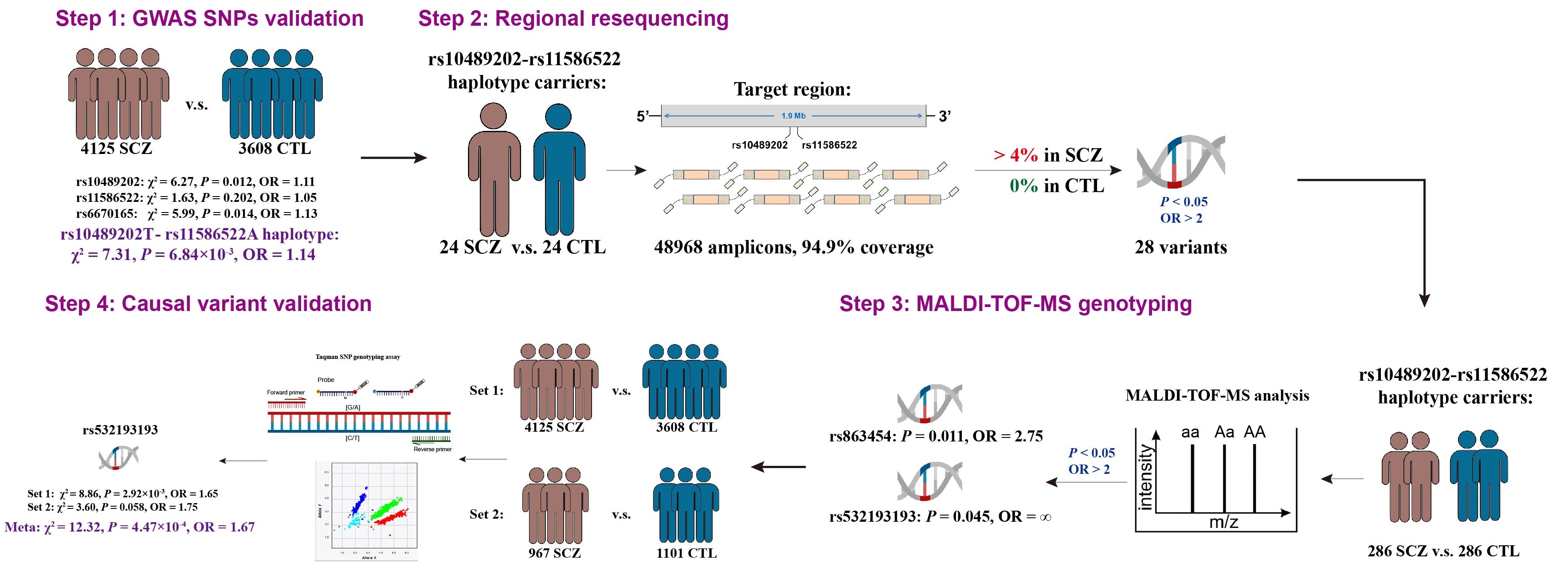


**Supplementary Figure 1.** A schematic illustration of research strategy for identification of a causal variant for schizophrenia. Step 1 to identify a schizophrenia-associated rs10489202-rs11586522 haplotype, step 2 to identify disease-related variants by deep sequencing of a 2-Mb region surrounding rs10489202 and rs11586522 in homozygotic case-control samples that carried two copies of the schizophrenia-associated rs10489202-rs11586522 haplotype, step 3 to verify a possible causal variant in a large mixed case-control sample and step 4 to verify the
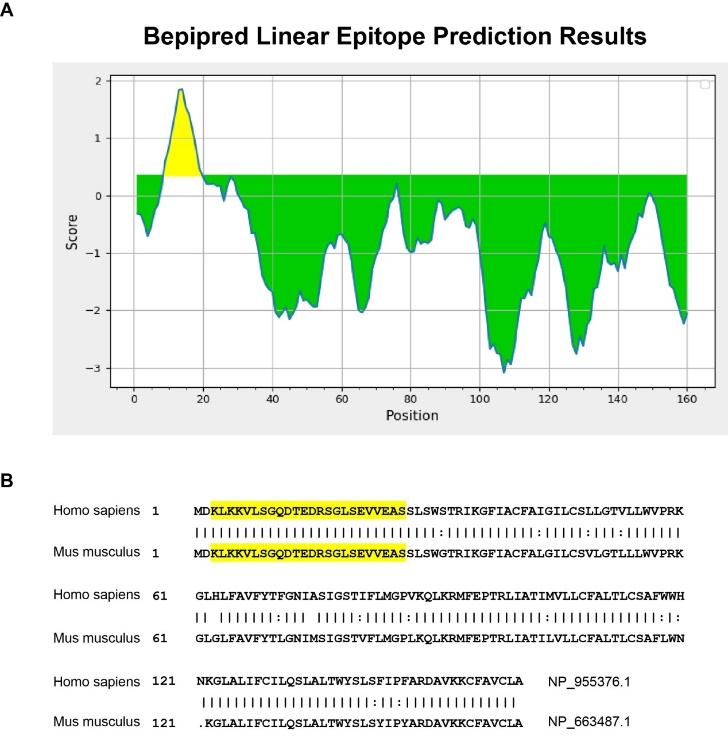
causal variant in an independent sample

**Supplementary Figure 2.** B cell epitope prediction of SFT2D2. **(A).** Antigen sequence properties of SFT2D2 protein analyzed by Bepipred Linear Epitope Prediction tool (http://tools.iedb.org/bcell/). The residues with scores above the threshold (default value is 0.35) are predicted to be part of an epitope marked in yellow on the graph. **(B).** Homology comparison of human and mouse protein sequences of SFT2D2. There was 91.19% sequence homology between human and mice, with the immunizing peptide being 100% homologous colored in yellow.

**
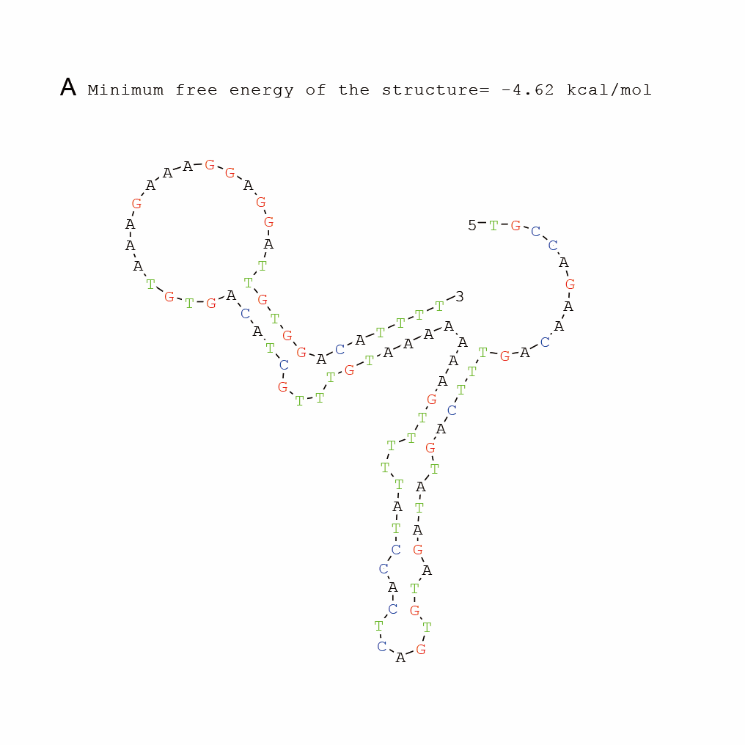
**

**
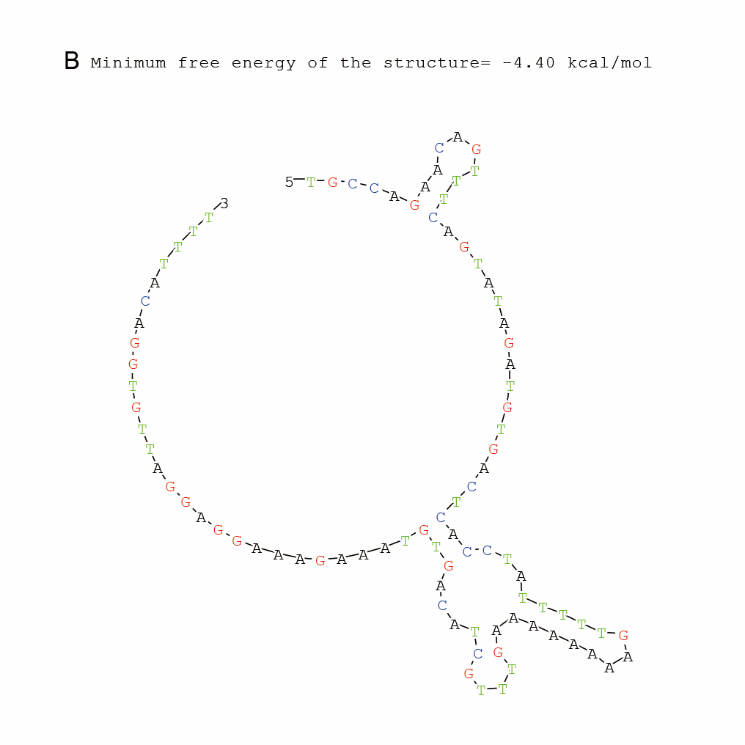
**

**Supplementary Figure 3.** Simulation of the DNA secondary structures. **(A).** The DNA secondary structure formed from the rs532193193T allele containing sequence; **(B).** The DNA secondary structure formed from the rs532193193A allele containing sequence. The DNA sequences used for simulation of the DNA secondary structures are as follow:

TGCCAGAACAGTTTCAGTATAGATGTGACTCACCTATTTTTGAAAAAAA[T/A]GTTTGCTACAGTGTAAAGAAAGGAGGATTGTGGACATTTT


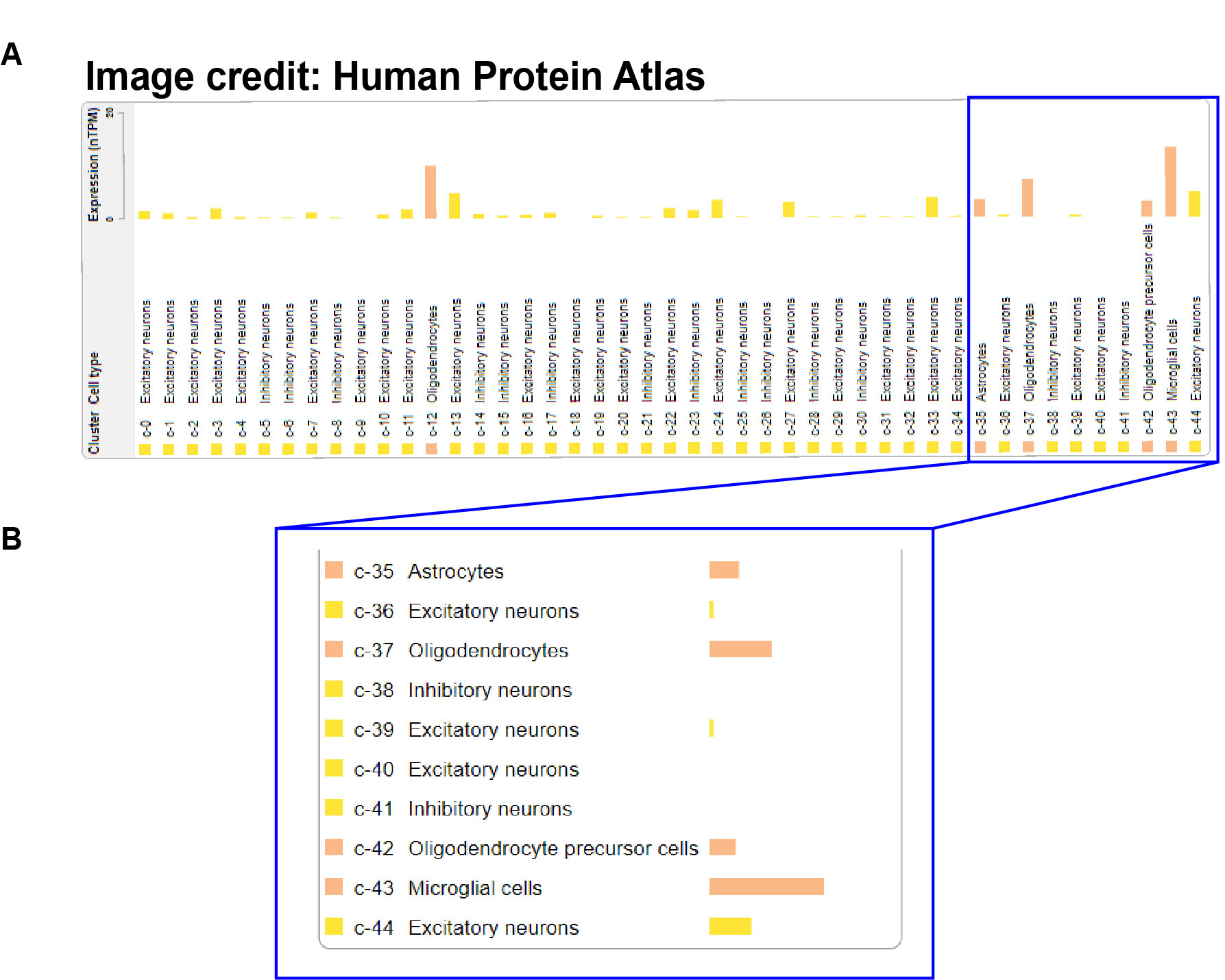


**Supplementary Figure 4. (A).** *SFT2D2* mRNA levels in single cell sequencing of brain cells from Human Protein Atlas (<https://www.proteinatlas.org/ENSG00000213064-SFT2D2/single+cell+type/brain>). **(B).** Enlargement of the blue framed inset in (A).


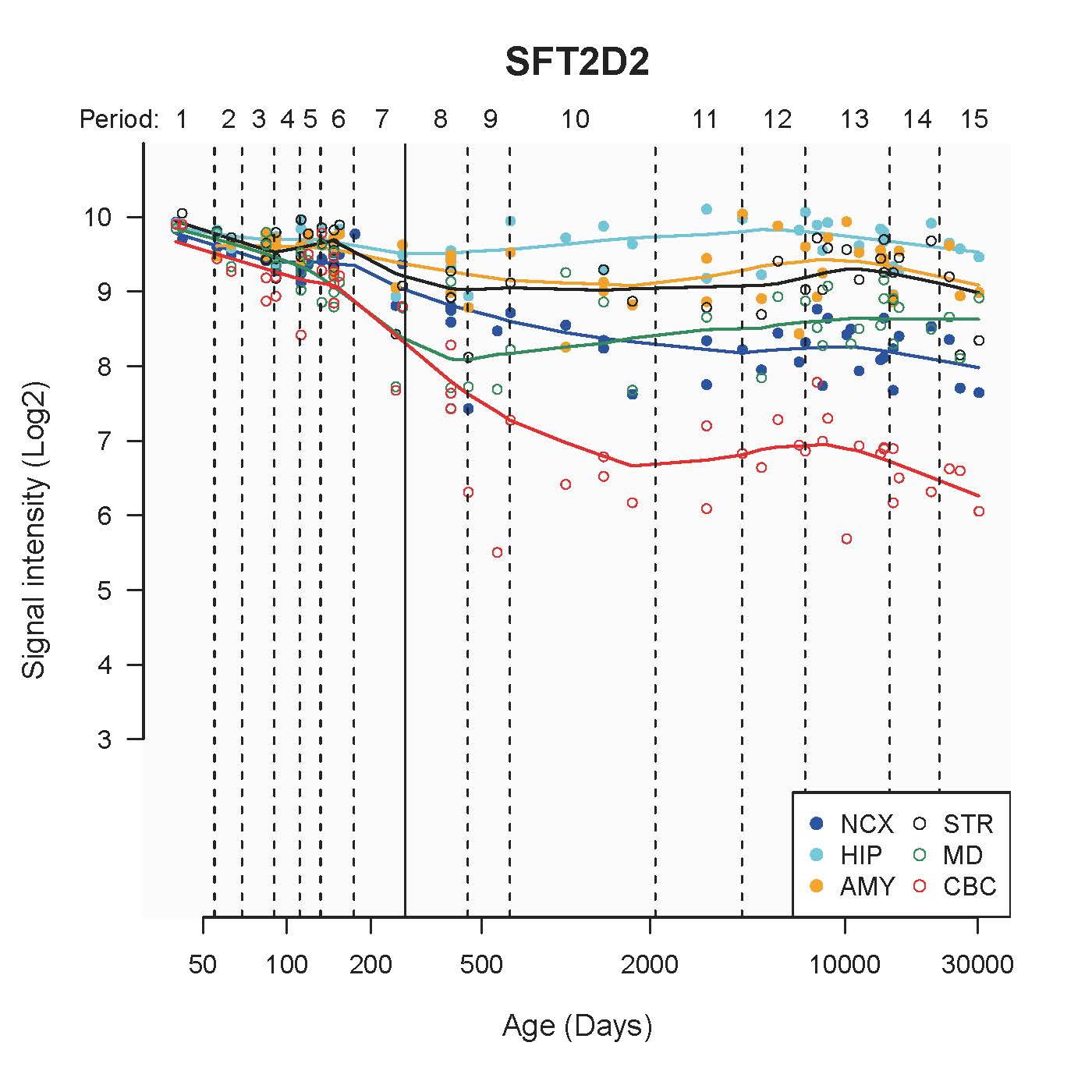


**Supplementary Figure 5.** Expression level of *SFT2D2* gene across the entire developing stages (from 8 post-conception weeks to 80 years) were depicted in the brain regions, which was divided into six regions: neocortex (NCX), hippocampus (HIP), amygdala (AMY), striatum (STR), thalamus (MD) and cerebellar cortex (CBC).

## Supplementary Tables

**Supplementary Table 1. Demographic and medication history**

|  | **Set1 （DNA）** | | **Set2 (DNA）** | | **Set 3（Plasma）** | |
| --- | --- | --- | --- | --- | --- | --- |
|  | **CTL**  **(*n* =3608)** | **SCZ**  **（n=4125）** | **CTL**  **(*n* =1011 )** | **SCZ**  **（n=967）** | **CTL**  **(*n* =350 )** | **SCZ**  **（n=332）** |
| Age (y) | 41.6±14.1 | 37.9±12.4 | 33.4±6.5 | 38.3±12.1 | 40.0±9.6 | 35.2±12.2 |
| Male/Female | 2556/ 1569 | 1467/ 2141 | 576/ 435 | 483/ 484 | 165/ 185 | 207/ 125 |
| Education (y) | 14.2±5.4 | 13.1±3.5 | 15.4±4.5 | 11.2±3.3 | 16.3±2.7 | 12.1±1.8 |
| Length of illness (y) | NA | 5.8±4.6 | NA | 6.4±3.8 | NA | 4.3±2.5 |
| History of antipsychotic drug use (n) |  |  |  |  |  |  |
| Clozapine | NA | 1125 | NA | 214 | NA | NA |
| Olanzapine | NA | 2789 | NA | 578 | NA | NA |
| Risperidone | NA | 2169 | NA | 648 | NA | NA |

Abbreviations: CTL, control; SCZ, schizophrenia. Values are shown as mean ± SD.

**Supplementary Table 2. Inclusion and exclusion criteria of participants**

| **Inclusion criteria for patients with schizophrenia:** |
| --- |
| (1). Clinical diagnosis was made by at least two senior psychiatrists according to the Diagnostic and Statistical Manual of mental disorders, fourth edition (DSM-IV) for schizophrenia. |
| (2). Chinese. |
| **Exclusion criteria for patients with schizophrenia:** |
| (1). Suffered from any organic brain disorders, mental retardation, epilepsy, head trauma. |
| (2). Showed psychotic symptoms due to medical conditions or treatments. |
| (3). Have a history of head trauma and loss of consciousness for more than 30 minutes |
| (4). Dependence on or abuse of alcohol or other substances. |
| (5). Obvious mental retardation. |
| (6). Abnormal biochemical indexes or abnormal EEG and ECG were found in physical examination or laboratory examination. |
| (7). Pregnant or lactating women. |
| **Inclusion criteria for healthy controls:** |
| (1). The healthy controls were recruited from local communities. |
| (2). The sex, age, native place and education of the healthy controls were matched with the patient group |
| (3). No smoking, drinking, staying up late, etc. within a week before blood collection |
| **Exclusion criteria for healthy controls:** |
| (1). Have history of major psychiatric or neurological disorders. |
| (2). Had family history of severe forms of psychiatric disorders. |
| (3). Febrile convulsions have occurred in children or infants. |
| (4). Adopted or living in a single parent family before the age of 18. |

**Supplementary Table 3. Information of primers used for MALDI-TOF-MS genotyping of twenty-eight variations in chromosomal 1q24.2 region**

| **position** | **Variation** | **1st-PCRP** | **2nd-PCRP** |
| --- | --- | --- | --- |
| 167242634 | rs374525798 | ACGTTGGATGGCCTCATGCAAAACCTCTAC | ACGTTGGATGTCTGTGGCGACTGTGTGTG |
| 167343488 | rs532966862 | ACGTTGGATGTGCTGCTGGAGCCACCATCT | ACGTTGGATGCGATCTGTATGGGCTGAGAC |
| 167458047 |  | ACGTTGGATGTATTCTCTCTGATGGAGCCC | ACGTTGGATGACTCACGTACGTGACATAAA |
| 167475871 | rs1799704 | ACGTTGGATGCAATATCGTGATTAGGCTGG | ACGTTGGATGCAAGGCTTGCTGTCTACTCT |
| 167475885 | rs7534569 | ACGTTGGATGTGAAGGTAGAACTAAGTGGC | ACGTTGGATGCCTGTGGCAATATCGTGATT |
| 167482045 | rs858551 | ACGTTGGATGCCATCTCACAGAGGAAGAAC | ACGTTGGATGACTATGGTGGAAGGAGAGG |
| 167488799 | rs863454 | ACGTTGGATGGTACCAAGTCTGATGATGGG | ACGTTGGATGTGAAGAAAGTGAGTCCTGC |
| 167562802 | rs548135867 | ACGTTGGATGTAACCAAGGCAGCACTTGAG | ACGTTGGATGCCAAGCAGCACAGAAGAGTA |
| 167571678 | rs539465026 | ACGTTGGATGCGTGAAACAGGTCATTATG | ACGTTGGATGCAGCTCTTGGAATGAATAAA |
| 167743526 | rs568146973 | ACGTTGGATGACTCCAGCTGGAAAGCTAAC | ACGTTGGATGACCAGCAAGAGAGTTCAGG |
| 167825485 | rs2071921 | ACGTTGGATGAGAACAGGAACACCACCTAC | ACGTTGGATGATGCAGCTCACATTGAGGTC |
| 167863359 | rs532020960 | ACGTTGGATGGCCAGGGGAAATCCAAAGAG | ACGTTGGATGGGTTTTGAGCTACATGTGAC |
| 167923123 |  | ACGTTGGATGAGCAGAGAACAAAGGTATG | ACGTTGGATGCTCCAAAAGTGCTGGGATTG |
| 167935501 |  | ACGTTGGATGCGTGTGTTAGACAAGCTTGA | ACGTTGGATGGCTAAGCTTCTTACGTGTGG |
| 167993356 |  | ACGTTGGATGGGATCACTTGAACCCAGAAA | ACGTTGGATGACTGGATCCTTGAACTCCTG |
| 168095936 | rs564160350 | ACGTTGGATGTCCCATCAGTTTGAGCAGCC | ACGTTGGATGAACTCTTGGACTCAGGTGAC |
| 168117597 | rs533350603 | ACGTTGGATGAAATTAGCCAGGTGTGGTAG | ACGTTGGATGCTGTGTGGTTATTCTACAGT |
| 168206831 | rs532193193 | ACGTTGGATGGTATCTGCCAGAACAGTTTC | ACGTTGGATGCACAATCCTCCTTTCTTTAC |
| 168207770 |  | ACGTTGGATGTGGACAGTGTAGCAAGACCG | ACGTTGGATGGCTCAAGTCTAGGAAGCTCA |
| 168213437 |  | ACGTTGGATGAAAAATTAGCACGGGCGTGG | ACGTTGGATGCTGTGGCTAAGAACTCTGAC |
| 168251318 | rs566388016 | ACGTTGGATGAGCTGAATGTTGGAGCCAGT | ACGTTGGATGTCACTTCGTAGAGGAGACGC |
| 168259441 | rs529952842 | ACGTTGGATGAGTAACTGCTAATGGCCGTG | ACGTTGGATGGCTGGCTTATAAGTCTAGAG |
| 168302624 | rs571065842 | ACGTTGGATGGTCTAAAAAGGGAGGCATGA | ACGTTGGATGCACCCATAGAGCTGCTGGTT |
| 168402778 |  | ACGTTGGATGTGCCACTTGCCAGGAAGGTT | ACGTTGGATGACAGGGTACTCACAGAGATG |
| 168603791 | rs528912102 | ACGTTGGATGGTCAGTGAAGGAGTGAGGAA | ACGTTGGATGTGAACACGATCAGCCCTGTC |
| 168722434 |  | ACGTTGGATGGTGTTGGTGGGAATGGTTC | ACGTTGGATGATCCTCCATGAGCGCAGCA |
| 168122619 | rs543569784 | TCCCGGCCTTATCTTATTC | AAGTAGTTCTTTGGGCTGGTC |
| 168262071 | rs377343745 | GAAGGAGCAGTTTGTTACCATT | CCTGTAATCCCAGCACTTTG |

**Supplementary Table 4. Information of primers and probes used for genotyping of rs10489202, rs11586522, rs863454, rs532193193 and rs6670165**

| **Name** | | **Sequence** | **Base** | **MW** | **GC%** | **Tm (℃)** |
| --- | --- | --- | --- | --- | --- | --- |
| rs10489202-F | CATTTCTACACTGGTGTGCCTCC | | 23 | 6942 | 52.2 | 57.1 |
| rs10489202-R | TGTTGTGAAAATCAGATGATCTAATGTCT | | 29 | 8946 | 31.0 | 54.4 |
| rs10489202-P1 | CCTTACTCTGTATGTTGCT | | 19 | 7413 | 42.1 | 67.0 |
| rs10489202-P2 | CCTTACTCTTTATGTTGCTC | | 20 | 7884 | 40.0 | 67.0 |
| rs11586522-F | GCACATTAGGCAGGGATTTTTC | | 22 | 6765 | 45.5 | 53.0 |
| rs11586522-R | GCTTCCTCAAGCCTGCTCTG | | 20 | 6020 | 60.0 | 55.9 |
| rs11586522-P1 | ACCACGTCATTCCCTCCGTGG | | 21 | 7409 | 61.9 | 60.6 |
| rs11586522-P2 | ACCACGTCATTACCTCCGTGGGA | | 23 | 8283 | 56.5 | 61.0 |
| rs863454-F | TTGTGCATCAAAGGACACTATGAA | | 24 | 7385 | 37.5 | 58.3 |
| rs863454-R | CTGATGATGGGAATGCTGTTTC | | 22 | 6797 | 45.5 | 58.1 |
| rs863454-P1 | TGAGTCCTGCAAATCACCGCATACC | | 25 | 8662 | 52.0 | 66.8 |
| rs863454-P2 | TGAGTCCTGCAAATCAACGCATACC | | 25 | 8893 | 48.0 | 65.0 |
| rs532193193-F | AATGTCCACAATCCTCCTTTCTTT | | 24 | 7189 | 37.5 | 52.3 |
| rs532193193-R | GGGATGGTATCTGCCAGAACAG | | 22 | 6825 | 54.5 | 56.7 |
| rs532193193-P1 | ACTGTAGCAAACTTT | | 15 | 6228 | 33.3 | 33.7 |
| rs532193193-P2 | ACTGTAGCAAACATT | | 15 | 6375 | 33.3 | 33.7 |
| rs6670165-F | AGGCTTAATTTTATTTTTAACTAAGAAGGTTCTACA | | 36 | 11062 | 25.0 | 56.5 |
| rs6670165-R | CTGTGACCCCCTGCTCAATAG | | 21 | 6342 | 57.1 | 56.3 |

**Supplementary Table 5. Analysis of plasma anti-SFT2D2 IgG against a linear antigen**

|  | **SBR, Mean (SD)** | |  |  |  |  |  |  | **Asymptotic 95% CI** | |
| --- | --- | --- | --- | --- | --- | --- | --- | --- | --- | --- |
|  | **Control** | **Schizophrenia** | **Fold Change** | ***Z*** | ***P*^a^** | **Sensitivity (%)^b^** | **AUC** | **SE** | **Lower** | **Upper** |
| **Total** | 0.391(0.045) | 0.722(0.174) | 1.847 | -13.686 | <0.0001 | 28.57 | 0.803 | 0.017 | 0.770 | 0.836 |
| **Male** | 0.362(0.040) | 0.718(0.208) | 1.983 | -10.572 | <0.0001 | 39.13 | 0.819 | 0.021 | 0.776 | 0.862 |
| **Female** | 0.417(0.048) | 0.729(0.119) | 1.748 | -8.957 | <0.0001 | 29.60 | 0.800 | 0.026 | 0.748 | 0.852 |

^a^ *P* < 0.05 was considered to be statistically significant.

^b^ Against a specificity of 95%.
